# Heart ventricular function in hospitalized patients with severe hypothyroidism and myxedema coma

**DOI:** 10.1210/clinem/dgag036

**Published:** 2026-01-30

**Authors:** Joaquin Lado-Abeal, Irsa Munir, Khalid Al-Zubaidi, Sobrina Mohammed, Shourya Tadisina, Miranda J Stiewig-Rapp, Aliakbar Arvandi, Ty Whisenant, Maria Jose Ginzo-Villamayor, Cristina Tejera-Perez, Patricia Perez-Castro, Eva Fernandez-Rodriguez, Aili Guo, Prasanth Surampudi, Rajib Bhattacharya, Yang Kevin Xiang, Thomas W Smith, Antia Fernandez-Pombo

**Affiliations:** Division of Endocrinology, Diabetes and Metabolism, Department of Internal Medicine, University of California Davis Medical Center and School of Medicine, Sacramento, CA 95617, USA; Division of Cardiovascular Medicine, Department of Internal Medicine, University of California Davis Medical Center and School of Medicine, Sacramento, CA 95617, USA; Division of Endocrinology, Diabetes and Metabolism, Department of Internal Medicine, University of California Davis Medical Center and School of Medicine, Sacramento, CA 95617, USA; Division of Endocrinology, Diabetes and Metabolism, Department of Internal Medicine, University Health Truman Medical Center, University of Missouri—Kansas City School of Medicine, Kansas City, KS 64018, USA; Division of Endocrinology, Diabetes and Metabolism, Department of Internal Medicine, University Health Truman Medical Center, University of Missouri—Kansas City School of Medicine, Kansas City, KS 64018, USA; Division of Endocrinology, Diabetes and Metabolism, Department of Internal Medicine, University of California Davis Medical Center and School of Medicine, Sacramento, CA 95617, USA; Division of Cardiology, Department of Internal Medicine, Texas Tech University Health Sciences Center (TTHUSC) School of Medicine and University Medical Center, Lubbock, TX 79430, USA; Division of Cardiology, Department of Internal Medicine, Texas Tech University Health Sciences Center (TTHUSC) School of Medicine and University Medical Center, Lubbock, TX 79430, USA; Department of Statistics, Mathematical Analysis and Optimization, University of Santiago de Compostela, 15782 Santiago de Compostela, Spain; Division of Endocrinology and Nutrition, University Clinical Hospital of Ferrol, 15405 A Coruña, Spain; Division of Endocrinology and Nutrition, University Clinical Hospital of Vigo, 36312 Vigo, Spain; Division of Endocrinology and Nutrition, University Clinical Hospital of Ourense, 32005 Ourense, Spain; Division of Endocrinology, Diabetes and Metabolism, Department of Internal Medicine, University of California Davis Medical Center and School of Medicine, Sacramento, CA 95617, USA; Division of Endocrinology, Diabetes and Metabolism, Department of Internal Medicine, University of California Davis Medical Center and School of Medicine, Sacramento, CA 95617, USA; Division of Endocrinology, Diabetes and Metabolism, Department of Internal Medicine, University Health Truman Medical Center, University of Missouri—Kansas City School of Medicine, Kansas City, KS 64018, USA; Department of Pharmacology, University of California Davis School of Medicine, Davis, CA 95616, USA; Division of Cardiovascular Medicine, Department of Internal Medicine, University of California Davis Medical Center and School of Medicine, Sacramento, CA 95617, USA; Division of Endocrinology and Nutrition, University Clinical Hospital of Santiago, University of Santiago de Compostela School of Medicine, 15706 Santiago de Compostela, Spain

**Keywords:** hypothyroidism, myxedema coma, echocardiogram, speckle-tracking echocardiogram (STE), global longitudinal strain (GLS), left ventricular ejection fraction (LVEF)

## Abstract

**Context:**

The clinical relevance of thyroid hormone–mediated inotropic effects in humans remains uncertain.

**Objective:**

We evaluated ventricular function in patients with severe hypothyroidism and myxedema coma using transthoracic echocardiography and speckle-tracking echocardiography.

**Methods:**

Multicenter, retrospective cross-sectional study of adults admitted to intensive care units with severe hypothyroidism who underwent transthoracic echocardiography during hospitalization. Left ventricular (LV) systolic and diastolic function and myocardial deformation, assessed by global longitudinal strain (GLS), were analyzed.

**Results:**

One hundred and twelve patients were included (mean age 53.9 ± 13.7 years; 64.3% female). Myxedema coma was present in 33 patients (29.5%), and 13 (11.6%) had severe hypothyroidism with altered mental status without hypothermia or bradycardia. Median admission thyroid-stimulating hormone was 113.9 (41.9-500.0) mIU/L. The mean LV ejection fraction (LVEF) was 53.9 ± 13.7%, LV GLS −13.5 ± 4.2% (reference −24% to −16%), and right ventricular (RV) GLS −17.9 ± 6% (reference −35% to −17%). Reduced LVEF occurred in 37.5% of patients, abnormal LV diastolic function in 66.7%, and impaired LV and RV GLS in 68.2% and 34.0%, respectively. In multivariable models, β-blocker treatment was associated with lower LVEF and worse biventricular GLS; angiotensin-converting enzyme inhibitor/angiotensin receptor blocker treatment with lower LVEF; and calcium channel blocker exposure was associated with improved RV GLS. In-hospital mortality was 4.5% and occurred in patients with preserved LVEF.

**Conclusion:**

Severe hypothyroidism is associated with frequent systolic and diastolic ventricular dysfunction and prevalent impairment of myocardial deformation. Associations between cardiovascular medication exposure and echocardiographic abnormalities likely reflect confounding by indication and underlying clinical phenotype rather than reflecting causal drug effects. Despite substantial myocardial abnormalities, short-term mortality was low.

Thyroid hormones (THs) regulate cardiac electromechanical activity (1-3). Although their chronotropic effects are well established, the clinical relevance of their inotropic actions remains uncertain (4-8). Patients with hypothyroidism commonly exhibit a prolonged pre-ejection period, delayed isovolumetric contraction and relaxation times, and slowed mitral inflow deceleration, an indicator of impaired left ventricular (LV) relaxation, all of which may have clinical implications, particularly in the setting of heart failure (8).

Thyroid hormone modulates cardiac function through multiple mechanisms, including regulation of adrenergic responsiveness (9-12), excitation-contraction (EC) coupling (13), myocardial energy metabolism (14), and systemic vascular resistance, which indirectly influences myocardial contractility via afterload (10). Case reports of patients with severe hypothyroidism presenting with congestive heart failure have shown improvement with TH replacement therapy (15, 16). However, studies using echocardiography and cardiac nuclear magnetic resonance to assess cardiac contractility in hypothyroid patients have found preserved or only mildly reduced LV ejection fraction (LVEF) (17-20), raising questions about the true impact of hypothyroidism on myocardial performance.

Speckle-tracking echocardiography (STE) allows quantification of myocardial strain, a dimensionless index of ventricular myocardial deformation that reflects the relative change in myocardial length during the cardiac cycle (21). Because longitudinal myocardial shortening results in negative strain values, less negative values indicate impaired contractility. Myocardial strain imaging is a sensitive and reproducible marker of myocardial dysfunction (22), and global longitudinal strain (GLS) is the gold standard for LV strain analysis (23), widely used to evaluate systolic function (24). Global longitudinal strain has demonstrated superior predictive value than LVEF for heart failure hospitalization, acute coronary ischemic events, cardiac death, and all-cause mortality (25, 26).

Patients with severe biochemical and clinical hypothyroidism, including myxedema coma, represent a unique human model to assess the real-world cardiac consequences of prolonged thyroid hormone deficiency. Accordingly, this study aimed to evaluate LV systolic function using transthoracic echocardiography (TTE) and GLS via STE in a large multicenter cohort of patients admitted to intensive care units (ICUs) with overt hypothyroidism.

## Materials and methods

### Study design and population

**Figure 1:**
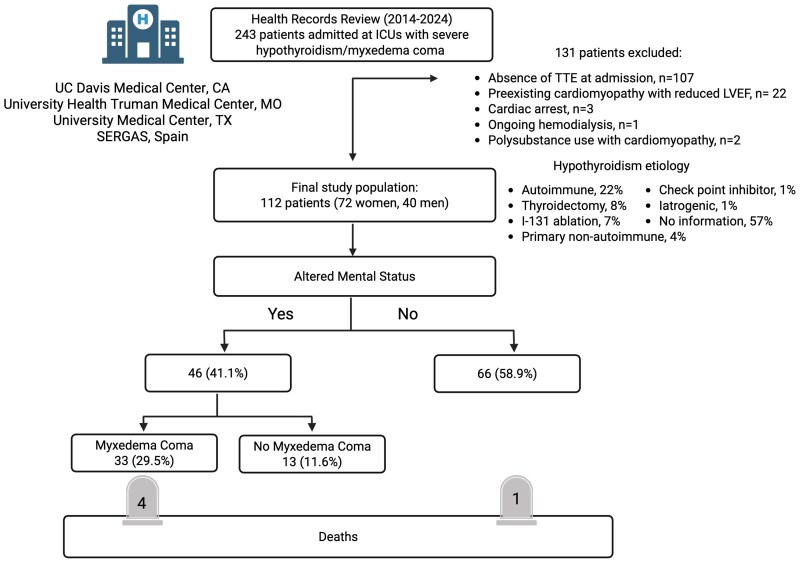
Study flow diagram. Electronic health records from the 4 tertiary participant centers were reviewed to identify ICU admissions for severe hypothyroidism or myxedema coma between 2014 and 2024. Of 243 eligible patients, 131 were excluded based on predefined criteria. The final cohort comprised 112 patients, stratified by the presence of an altered mental status and diagnosis of myxedema coma. In-hospital mortality is shown. Abbreviations: ICUs, intensive care units; LVEF, left ventricular ejection fraction; TTE, transthoracic echocardiography.

This study was a multicenter, retrospective, cross-sectional analysis of patients admitted to ICUs from 2014 to 2024 with overt hypothyroidism who underwent TTE during hospitalization (Fig. 1). Participating centers included the University of California Davis Medical Center (Sacramento, CA; *n* = 68), University Health Truman Medical Center (Kansas City, MO; *n* = 19), University Medical Center (Lubbock, TX; *n* = 16), and the Servicio Galego de Saude (SERGAS) Hospitals Network (Galicia, Spain; *n* = 9).

Clinical and biochemical data were obtained from electronic health records. At each center, TTEs were reviewed by the same board-certified cardiologists with expertise in echocardiography. Among 243 adult patients (≥18 years) initially identified, 131 were excluded for one or more of the following reasons: absence of TTE at admission (*n* = 107), preexisting cardiomyopathy with reduced LVEF (*n* = 22), cardiac arrest (*n* = 3), ongoing hemodialysis (*n* = 1), or polysubstance use (*n* = 2; Fig. 1). Additional exclusion criteria included implanted cardiac devices, atrial fibrillation, active amiodarone therapy, prior cardiotoxic chemotherapy, urgent surgery, or septic shock upon admission; none were present in the final cohort. The study population, therefore, comprised 112 patients.

The study was approved by the Institutional Review Boards of all participating centers and conducted in accordance with the Declaration of Helsinki.

### Clinical and biochemical data collection

Demographic, anthropometric, clinical, biochemical, and medication variables were recorded at the time of TTE. Biochemical analysis included serum thyroid-stimulating hormone (TSH; mUI/L), free T4 (ng/dL), sodium, potassium, calcium, chloride, and total carbon dioxide (mmol/L).

Thyroid hormone measurements were performed at the central laboratories at each institution, using standardized immunoassays, with minimal inter-center variability. Assay platforms included a Bio-Rad immunoassay plus QC on a Beckman Coulter IniCell Dxl 800 immunoassay analyzer, Roche Cobas analytical systems, and Siemens ADVIA Atellica analyzers (27).

Myxedema coma was diagnosed in patients with altered mental status, hypothermia (temperature <36 °C), and bradycardia (heart rate <60 beats per minute) in the setting of severe biochemical hypothyroidism (TSH >40 mUI/L) and in the absence of an alternative explanation (28).

### TTE and speckle-tracking analysis

All patients underwent a TTE. Image acquisition and analysis were performed in accordance with the American Society of Echocardiography and the European Association of Cardiovascular Imaging (23).

Left ventricular ejection fraction was measured using 2DE TTE using Simpson's biplane method in 2- and 4-chamber views. Reduced LVEF was defined as <52% in males and <54% in females, and was categorized as mild 41% to 51% in males, 41% to 53% in females, moderate 30% to 40%, and severe <30% (23).

Left ventricular diastolic function (LVDF) was assessed using pulsed-wave Doppler mitral inflow, tissue Doppler imaging of the mitral annulus, left atrial volume index (biplane disk method), and tricuspid regurgitation velocity. For analysis, LVDF was classified as normal, abnormal (grades I-III), or indeterminate (29).

**Figure 2:**
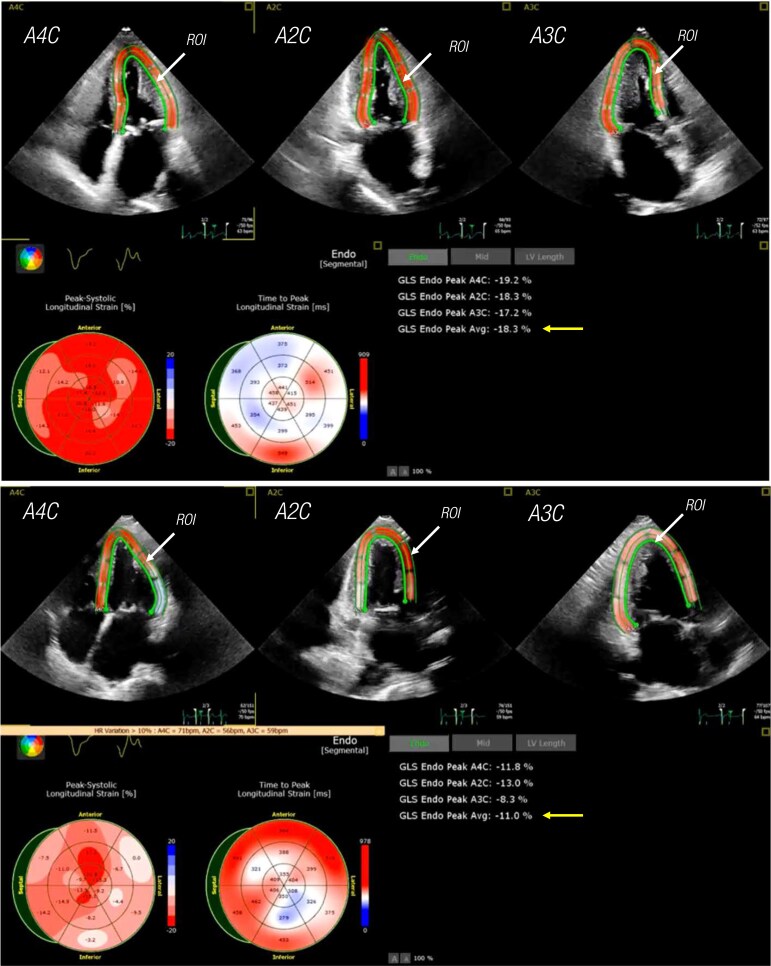
Global longitudinal strain (GLS) assessed by speckle-tracking echocardiography (STE). Global longitudinal strain was derived from apical 4-chamber (A4C), 2-chamber (A2C), and 3-chamber (A3C; apical long-axis) views. The myocardial region of interest (ROI) was defined by endocardial and epicardial contours. The bull's-eye (polar) plot summarizes peak systolic longitudinal strain, with central, middle, and outer rings corresponding to apical, mid-ventricular, and basal segments, across inferior, anterior, lateral, and septal regions. More negative strain values indicate better myocardial contractility. Color coding reflects strain magnitude (red/dark = more negative, better strain; yellow/green = intermediate; and blue/pale = less negative, worse strain). Diffuse color attenuation suggests global left ventricular dysfunction, whereas focal blue regions indicate regional dysfunction. The numeric GLS value represents the average strain across all segments. Abnormal GLS was defined as >−16% for the left ventricle (LV) and > −17% for the right ventricle. Representative examples show a patient with preserved LV GLS (−18.3%; upper panel) and a patient with markedly reduced GLS (−11%) consistent with severe LV systolic dysfunction (lower panel).

Global longitudinal strain was quantified from standard 2D images using the Tomtec multimodality imaging system (30) (Fig. 2). High-quality apical 4-, 2-, and 3-chamber DICOM images with adequate frame rates (>15 frames/second) and no apical foreshortening were selected. Automated endocardial tracking was manually adjusted when necessary and independently verified by 2 experienced cardiologists. Papillary muscles, trabeculations, and the pericardium were excluded from the region of interest. A similar methodology was applied for right ventricular (RV) strain analysis using RV-focused apical 4-chamber views. Left ventricular GLS (normal range, −24% to −16%) was available in 85 patients, and RV GLS (normal range, −35% to −17%) in 53 patients (31).

### Electrocardiography

A resting 12-lead electrocardiogram was obtained at admission. Heart rate, PR interval, QRS duration, QTc interval, and ST-T segment were analyzed. Prolonged intervals were defined as PR interval >200 ms, QRS complex >120 ms, and QTc interval ≥450 in males and ≥460 in females.

### Statistical analysis

Data are presented as the mean ± standard deviation (SD), median, and interquartile range (IQR) or as *n* (%) values. Normality was assessed using the Shapiro–Wilk test. Between-group comparisons used the Student's *t*-test or Mann–Whitney *U* test for continuous variables. Qualitative variables were compared using the χ^2^ or Fisher's exact tests. Correlations were assessed using Pearson's or Spearman's coefficients, as appropriate.

Multiple linear regression analysis was performed to examine the independent associations of medication (β-blockers, angiotensin-converting enzyme inhibitors [ACEi]/angiotensin receptor blockers [ARBs], calcium channel blockers [CCBs], glucocorticoids, vasopressors, and albuterol/ipratropium), and serum electrolyte levels (sodium, potassium, calcium, chloride, and total carbon dioxide) with the dependent variables LVEF, LV GLS, and RV GLS. A *P*-value of <.05 was considered statistically significant. All statistical analyses were conducted using the SPSS 22.0 program (Chicago, IL, USA).

## Results

The study cohort comprised 112 patients with a mean age of 53.9 ± 13.7 years, a predominance of females (*n* = 72; 64.3%; Fig. 1), and a median hospital stay of 6 days (range, 1-66 days).

Autoimmune thyroid disease was the most common frequently identified etiology of hypothyroidism (25 patients, 22.32%), followed by total thyroidectomy (*n* = 9, 8%), radioactive iodine ablation (*n* = 8, 7.1%), primary nonautoimmune hypothyroidism (*n* = 4, 3.6%), immune checkpoint inhibitor-related hypothyroidism (*n* = 1, 0.9%), and other iatrogenic causes (*n* = 1, 0.9%). The etiology of hypothyroidism could not be determined in 64 patients (57%; Fig. 1).

**Figure 3:**
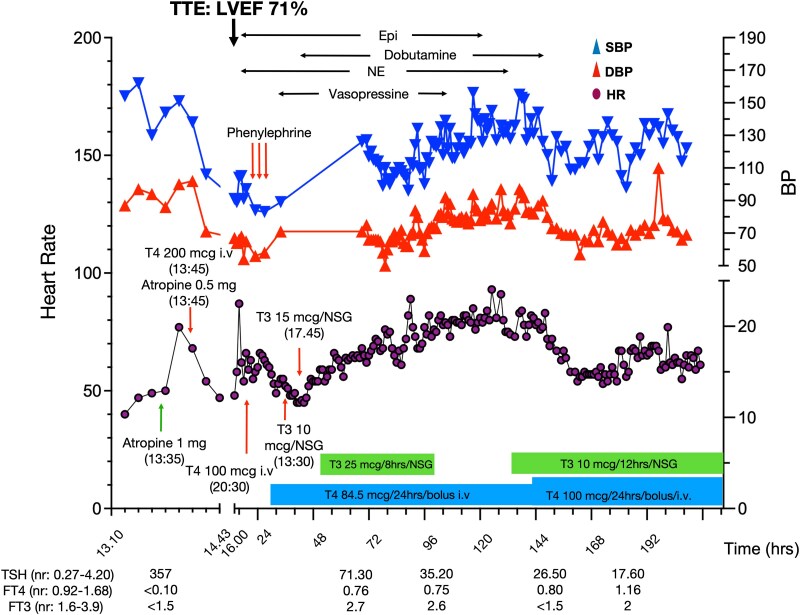
Hemodynamic course, organ support, and thyroid hormone replacement in a patient with myxedema coma. Heart rate, blood pressure, hemodynamic support, and thyroid hormone levels during the hospitalization are shown for a 74-year-old woman admitted with myxedema coma. At presentation, she exhibited bradycardia (40 beats per minute), hypothermia (35 °C), and hypertension, with preserved LVEF (71%). Heart rate increased following atropine administration, suggesting a predominant cholinergic chronotropic control. Approximately 2 hours after ICU admission, she developed hypotension and respiratory depression requiring endotracheal intubation and vasopressor support. Vasopressors were discontinued on hospital day 5, and extubation occurred on day 13. Thyroid hormone replacement consisted of daily intravenous levothyroxine, associated with a progressive TSH suppression and rising serum T4 levels; normalization of serum T3 required the addition of liothyronine administered via nasogastric tube. Abbreviations: DBP, diastolic blood pressure; Epi, epinephrine; HR, heart rate; LVEF, left ventricular ejection fraction; NE, norepinephrine; NSG, nasogastric; SBP, systolic blood pressure; TTE, transthoracic echocardiography.

Baseline anthropometric measures, clinical variables, and therapeutic interventions are summarized in Table 1, stratified by sex. Altered mental status was the most common presentation feature, observed in 46 subjects (41.1%). Of these, 33 met criteria for myxedema coma (29.5%), while 13 patients (11.6%) had severe biochemical hypothyroidism with altered mental status but without hypothermia or bradycardia (Fig. 1). Twelve patients (10.7%) had a history of coronary artery disease, none with previously documented reduced LVEF. Small pericardial effusions were frequently observed (*n* = 49; 46.2%). Prior to TTE, 77 patients (68.7%) received intravenous fluid resuscitation (mean volume, 2.3 L; range, 0.5-12.5 L). An illustrative example of hemodynamic changes and therapeutic interventions in a patient with myxedema coma is shown in Fig. 3.

**Table 1:** Cohort anthropometric measurement, clinical data, and therapeutical interventions

	Overall cohort		Female		Male		*P*-value
	*n*	Value	*n*	Value	*n*	Value	
Age (years)	112	55.8 ± 14.5	72	55.6 ± 14.4	40	56.3 ± 14.8	.942
BMI (kg/m^2^)	111	30.2 ± 8.8	71	30.5 ± 9.5	40	29.6 ± 7.5	.626
SBP at admission (mm Hg)	112	131.2 ± 33.7	72	130 ± 34.4	40	133.3 ± 32.7	.603
DBP at admission (mm Hg)	112	81.2 ± 20.7	72	78.5 ± 19.8	40	86.1 ± 21.8	.064
Heart rate at admission (bpm)	109	70.6 ± 17.3	72	70.9 ± 16.3	40	70 ± 19	.496
Pericardial effusion (*n*, %)	106	49 (46.2)	72	34 (47.2)	35	15 (42.9)	.036
Levothyroxine pre-TTE (*n*, %)	112	86 (76.8)	72	50 (69.4)	40	36 (90.0)	.026
Dose (μg)		150 (25-784)		127.5 (50-784)		200 (25-600)	.641
Liothyronine pre-TTE (*n*, %)	112	14 (12.5)	72	6 (5.4)	40	8 (7.1)	.086
Dose (μg)		10 (5-60)		12 (5-30)		10 (5-60)	.865
Other drugs administered							
Glucocorticoids (*n*, %)	112	53 (47.3)	72	32 (44.4)	40	21 (52.5)	.384
Vasopressors (*n*, %)	112	16 (14.3)	72	10 (13.9)	40	6 (15.0)	.386
Beta-blockers (*n*, %)	112	22 (19.6)	72	13 (18.1)	40	9 (22.5)	.625
ACEi or ARBs (*n*, %)	112	19 (17.0)	72	8 (11.1)	40	11 (27.5)	.140
Calcium antagonists (*n*, %)	112	19 (17.0)	72	14 (18.7)	40	5 (12.5)	.519
Albuterol/ipratropium (*n*, %)	112	28 (25.0)	72	20 (27.8)	40	8 (20.0)	.347
Hospitalization days	112	6 (1.0-66.0)	72	6 (1.0-66.0)	39	7 (2.0-43.0)	.594
Need for intubation (*n*, %)	112	20 (17.9)	72	11 (15.3)	40	9 (22.5)	.390
Death (*n*, %)	112	5 (4.5)	72	4 (5.6)	40	2 (5.0)	.722

Data are *n* (%), mean ± SD, or median (IQR) depending on normality.

Abbreviations: ACEi, angiotensin-converting enzyme inhibitor; ARBs, angiotensin receptor blockers; BMI, body mass index; bpm, beats per minute; DBP, diastolic blood pressure; SBP, systolic blood pressure; TTE, transthoracic echocardiogram.

### Thyroid function, treatment, and outcomes

**Figure 4:**
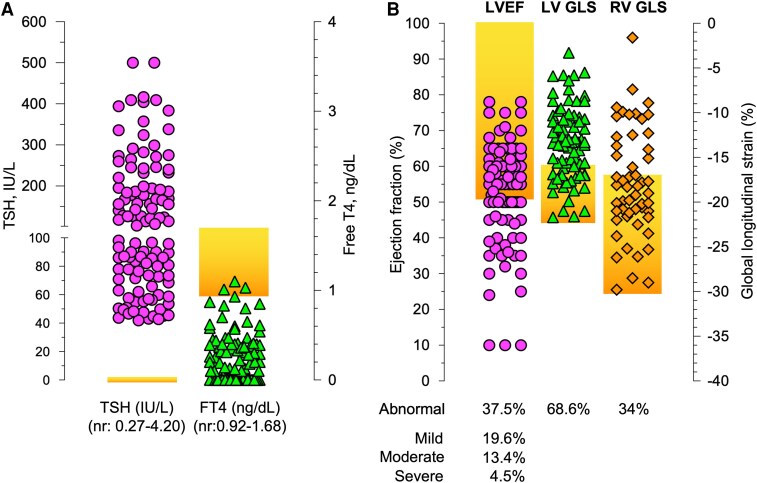
Thyroid hormone measurements and echocardiographic findings in patients with overt hypothyroidism admitted to the ICU. (A) Serum thyroid-stimulating hormone (TSH) and free thyroxine (FT4) concentrations in the study cohort (*n* = 112). (B) Left ventricular ejection fraction (LVEF; *n* = 111) and left (*n* = 85) and right (*n* = 53) ventricular global longitudinal strain (GLS) assessed by a transthoracic echocardiogram and STE. Reduced LVEF was observed in 38% of patients. Abnormal LV GLS and RV GLS were present in 69% and 34% of patients, respectively. Bars indicate reference ranges; dots represent individual patient values. Abbreviations: LV, left ventricle; RV, right ventricle.

At admission, the median serum TSH concentration was 113.9 mIU/L (range, 41.9-500.0 mIU/L), and the median FT4 was 0.11 ng/dL (Table 2, Fig. 4A). Thyroid-stimulating hormone, FT4, and FT3 concentrations did not differ significantly between patients with myxedema coma and those without. Levothyroxine was administered before TTE in 86 patients (76.8%) at a median dose of 150 μg (range, 25-784 μg), and 14 patients (12.5%) received concomitant liothyronine (median dose, 10 μg; range, 5-60 μg).

**Table 2:** Serum thyroid hormone levels, echocardiographic, and electrocardiogram parameters of the cohort

	Overall sample		Female		Male		*P*-value
	*n*	Value	*n*	Value	*n*	Value	
Biochemical data							
TSH (mIU/L)	112	113.9 (41.9-500.0)	75	117.7 (42.0-500.0)	40	118.7 (43.0-409.0)	.428
FT4 (ng/dL)	112	0.11 (0.0-1.0)	73	0.2 (0.0-1.1)	39	0.1 (0.0-1.0)	.032
TTE and STE							
LVEF (%)	111	53.9 ± 13.7	72	54.8 ± 14.0	39	52.3 ± 13.1	.475
LVDF	93		60		33		.948
Normal (*n*, %)		16 (17.2)		10 (16.7)		7 (18.2)	
Abnormal (*n*, %)		62 (66.7)		40 (66.7)		21 (63.6)	
Indeterminate (*n*, %)		15 (16.1)		10 (16.7)		5 (15.2)	
LV GLS (%)	85	−13.5 ± 4.2	60	−13.9 ± 4.0	25	−12.5 ± 4.4	.186
RV GLS (%)	53	−17.9 ± 6.0	36	−17.8 ± 6.2	17	−18.2 ± 5.5	.808
ECG results							
Heart rate (bpm)	109	70.6 ± 17.1	69	70.9 ± 16.3	40	70.0 ± 19.0	.642
PR interval (ms)	100	162.1 ± 26.2	63	162.1 ± 27.6	37	162.1 ± 24.0	.641
QRS complex (ms)	104	96.5 ± 18.0	65	92.3 ± 14.1	39	103.4 ± 21.7	.005
QTc interval (ms)	104	455.5 ± 51.2	65	456.7 ± 51.6	39	452.4 ± 51.1	.755
Prolonged PR (*n*, %)	100	6 (6)	63	5 (7.9)	37	1 (2.5)	.264
Prolonged QRS (*n*, %)	104	9 (8.7)	65	1 (1.5)	39	8 (20.5)	.004
Prolonged QTc (*n*, %)	104	45 (43.2)	65	28 (43.1)	39	17 (43.6)	.136

Data are *n* (%), mean ± SD, or median (IQR) depending on normality. Prolonged PR is defined as a PR interval >200 ms, prolonged QRS as a QRS complex >120 ms, and prolonged QTc as a QTc interval ≥450 in males and ≥460 in females.

Abbreviations: bpm, beats per minute; ECG, electrocardiogram; FT4, free T4; GLS, global longitudinal strain; LV, left ventricular; LVDF, left ventricular diastolic function; LVEF, left ventricular ejection fraction; ms, milliseconds; QTc, corrected QT; RV, right ventricular; STE, speckle-tracking echocardiography; TTE, transthoracic echocardiogram.

In-hospital mortality occurred in 5 patients (4.5%), 4 of whom had myxedema coma (12.1% of that subgroup). All who died had preserved LVEF, and none had known coronary artery disease.

### Echocardiographic findings

The median time from admission to TTE was 1 day (range, 0-21 days). Mean LVEF was 53.9 ± 13.7%. Reduced LVEF was observed in 42 patients (37.5%), including mild dysfunction in 19.6%, moderate in 13.4%, and severe in 4.5% (Fig. 4B). Left ventricular diastolic function was abnormal in 62 cases (66.7%) and indeterminate in 15 cases (16.1%; Table 2).

Mean LV GLS was −13.5 ± 4.2%, with abnormal values (≥16%) in 68.6% of patients (Fig. 4B). Mean RV GLS was −17.9 ± 6.0%, with abnormal values (≥17%) in 34.0% of cases. No significant differences were observed in LVEF, LV GLS, or RV GLS by sex or by the presence of myxedema coma.

Serum TSH levels did not differ between patients with preserved and reduced LVEF. However, there was a nonsignificant trend toward higher TSH concentrations in patients with impaired LV GLS compared with those with normal GLS (TSH: 150.9 ± 122.2 vs 93.9 ± 63.7 mIU/L; *P* = .073). Total fluid resuscitation volume was not associated with either reduced LVEF or impaired LV GLS.

Comparisons of LVEF and GLS by medication exposure are shown in Table 3. Patients treated with β-blockers had significantly lower LVEF and worse LV and RV GLS than untreated patients. Treatment with ACEis or ARBs was also associated with lower LVEF. In the unadjusted analysis, ipratropium/albuterol therapy was associated with higher LV GLS.

**Table 3:** Comparison of left ventricle ejection fraction and global longitudinal ventricular strain by medication exposure

	LVEF			LV GLS			RV GLS		
	No	Yes	*P-*value	No	Yes	*P-*value	No	Yes	*P-*value
β-Blockers	55.5 ± 12.3	45.8 ± 17.6	.021	−14.2 ± 4.0	−10.2 ± 3.7	.001	−18.9 ± 5.7	−15 ± 6.0	.043
ACEi/ARBs	55.2 ± 13.2	46.8 ± 15.4	.017	−13.6 ± 4.0	−12.6 ± 5.2	.056	−17.7 ± 6.1	−19.3 ± 5.1	.400
CCBs	53.7 ± 14.5	54.2 ± 11	.863	−13.9 ± 4.4	−11.9 ± 2.6	.021	−17.6 ± 6.5	−19.3 ± 4.1	.277
Albuterol/ipratropium	53.8 ± 14.2	52.9 ± 13.5	.719	−14 ± 4.2	−11.9 ± 3.9	.033	−18.4 ± 6.1	−16.9 ± 5.8	.370
GC	51.8 ± 13.9	56.2 ± 13.2	.092	−12.7 ± 4.5	−14.4 ± 3.7	.057	−17.9 ± 6.3	−17.9 ± 5.6	.990
Vasopressors	53.1 ± 13.7	56.5 ± 15.7	.179	−13.5 ± 4.2	−13.4 ± 3.2	.866	−18.0 ± 6.1	−16.1 ± 4.5	.464

Data mean ± SD.

Abbreviations: LV GLS, left ventricular global longitudinal strain; LVEF, left ventricular ejection fraction; RV GLS, right ventricular global longitudinal strain. Yes, indicate treatment with: β-blockers, beta-blockers; ACEi/ARBs, angiotensin-converting enzyme inhibitors/angiotensin receptor blockers; CCBs, calcium channel blockers; albuterol and ipratropium; GC, glucocorticoids; Vasopressors.

In multivariable regression analysis, age, sex, and serum TSH were not independently associated with LVEF or ventricular strain parameters. In contrast, β-blocker therapy was independently associated with lower LVEF (intercept = 53.47%; estimate −9.84; *P* = .004) and worse LV GLS (intercept = −14.07; estimate = 3.71; *P* = .003) and RV GLS (intercept = −17.89; estimate = 7.84; *P* = .001). Angiotensin-converting enzyme inhibitor/ARB use was independently associated with lower LVEF (estimate −7.22; *P* = .029). Conversely, calcium channel blocker therapy was independently associated with lower (more negative) RV GLS (estimate −6.65; *P* = .009), indicating better RV systolic performance. The association between albuterol/ipratropium use and LV GLS was no longer significant after adjustment (*P* = .119). No significant associations were identified between serum electrolyte levels and LVEF or GLS.

### Electrocardiography

Electrocardiographic abnormalities were present in approximately half of the cohort. QTc prolongation was the most frequent abnormality, observed in 45 cases (43.2%). Prolonged QRS duration occurred in 9 patients (8.7%), and PR interval prolongation in 6 patients (6.0%; Table 2). No ST-T segment abnormalities were identified.

Although TSH concentrations did not differ between patients with and without myxedema, those with myxedema coma had a significantly longer PR interval than the remainder of the cohort (176.4 ± 37.6 vs 158.3 ± 26.2 ms, *P* = .006), with no differences by sex.

## Discussion

The present dataset, encompassing a large cohort of patients with severe hypothyroidism and myxedema coma admitted to the ICU, showed a high prevalence of LV dysfunction. This dysfunction is characterized by impaired systolic performance (reduced LVEF), abnormal diastolic function (LVDF), and/or increased myocardial strain, as reflected by abnormal GLS (Fig. 2).

Nearly 40% of patients exhibited reduced LVEF, a finding consistent with a prior study using cardiac magnetic resonance imaging (CMRI)—considered the gold standard for noninvasive cardiac contractility measurement—in patients with postsurgical hypothyroidism (18). In contrast, earlier TTE reported preserved LVEF in hypothyroidism patients compared with controls (4, 7, 20). These discrepancies likely reflect differences in cohort characteristics, particularly the severity and duration of hormonal deprivation, as well as hemodynamic conditions such as preload and afterload, which substantially influence ventricular performance (32, 33).

Left ventricular GLS is more sensitive than LVEF for detecting subtle systolic dysfunction and is recognized as an early marker of myocardial impairment (34) (Fig. 2). Global longitudinal strain is also a strong prognostic indicator for heart failure events and arrhythmias across multiple cardiovascular conditions (34). Importantly, strain measurements derived from CMRI and STE demonstrate good reproducibility and correlation for both LV and RV systolic and diastolic parameters (34). Most prior studies evaluating GLS conclude that hypothyroidism, even in its subclinical form, is associated with impaired myocardial deformation (8, 20, 35, 36). Our findings support these observations by showing that severe hypothyroidism is frequently associated with reduced myocardial contractility.

Diastolic dysfunction was present in ∼2/3 of our cohort. Previous studies have similarly reported a high prevalence of diastolic abnormalities, even among patients with subclinical hypothyroidism (19, 37). Hypothyroidism has been associated with increased ventricular wall thickness, particularly of the septal wall, and these changes appear reversible with thyroid hormone replacement (18). Proposed mechanisms include alterations in the extracellular matrix, with increased accumulation of collagen and glycosaminoglycans, contributing to increased myocardial stiffness and impaired relaxation (2, 18).

Electrocardiographic abnormalities were also highly prevalent, affecting nearly half of patients. QTc prolongation was the most frequent finding, present in over 43% of cases. This observation is consistent with established evidence that hypothyroidism induces electrophysiological changes, particularly delayed ventricular repolarization mediated by reduced expression and function of potassium channels involved in phase 3 repolarization (1). Notably, patients with myxedema coma exhibited significantly longer PR intervals than those with severe hypothyroidism without coma, despite similar TSH levels. PR prolongation reflects atrioventricular nodal conduction delay and may be accentuated by profound metabolic derangement and autonomic dysfunction characteristic of myxedema coma. These findings suggest that selected electrocardiographic parameters may better reflect tissue-level hypothyroidism and systemic severity than circulating TSH alone, consistent with the recognized dissociation between serum TSH and peripheral tissue hypothyroid effects in critical illness.

The mortality rate among patients with myxedema coma in our series (12%) aligns with previously reported estimates (38). Importantly, all patients with myxedema coma who died had preserved LVEF. Overall ICU mortality in the entire cohort was low (4.5%) and lower than reported mortality rates for general medical ICUs (10-15%). Thus, despite marked myocardial dysfunction, short-term mortality was not increased. Large population-based studies have shown that even subclinical hypothyroidism increases the risk of heart failure and cardiovascular mortality, particularly in patients with ischemic heart disease or impaired LV function (39, 40). In such settings, severe hypothyroidism may further exacerbate hemodynamic compromise, impair myocardial perfusion, and increase arrhythmogenic risk (41). To isolate the independent effects of severe hypothyroidism on myocardial performance, our study excluded patients with known cardiovascular disease or preexisting reduced LVEF, which may be essential modifiers of prognosis in hypothyroid patients (42). It is well recognized that hypothyroidism exerts more detrimental effects when superimposed on structurally compromised hearts, where reduced cardiac reserve limits the ability to compensate for additional hemodynamic stress (43). In our cohort, patients without overt structural heart disease may retain sufficient cardiac reserve to compensate for the reversible functional impairment induced by severe hypothyroidism. The impact of severe hypothyroidism in patients with established cardiovascular disease—a common comorbidity in hypothyroid populations—remains an important area for future investigation.

The myocardial abnormalities observed in hypothyroidism, including impaired GLS, diastolic dysfunction, and QT prolongation, likely reflect alterations in EC coupling rather than irreversible myocardial injury. Unlike structural heart disease, these abnormalities are potentially reversible with thyroid hormone replacement. Prompt initiation of thyroid hormone therapy and aggressive supportive ICU care may therefore mitigate adverse cardiac consequences. Notably, our mortality analysis was limited to short-term ICU outcomes; longer-term follow-up will be required to fully define the prognostic implications of myocardial dysfunction in this population.

In multivariable analyses, treatment with β-blockers and ACEi/ARBS was independently associated with lower LVEF and worse myocardial strain parameters. Given the observational, cross-sectional design, these associations should not be interpreted as causal. These medications are known to improve survival in patients with heart failure and reduced ejection fraction, with greater benefit observed in patients with more severe LVEF reduction and higher GLS (44, 45). However, therapeutic response is heterogeneous and influenced by etiology, neurohormonal activation, baseline hemodynamics, rhythm, myocardial reversibility, and comorbidities (44, 45). Severe hypothyroidism represents a distinct low-output state characterized by bradycardia, reduced inotropy, increased systemic vascular resistance, and impaired relaxation, in which the conventional neurohormonal model underlying these therapies may be less applicable. Supporting this concept, we previously demonstrated in an *in vitro* model of septic cardiomyopathy that triiodothyronine prevents tumor necrosis factor-α-induced β-adrenergic receptor desensitization, restoring EC coupling in cardiomyocytes (46). Altered thyroid hormone signaling may therefore modulate adrenergic responsiveness and myocardial mechanics, influencing echocardiographic measures of systolic function and strain in patients receiving standard heart failure therapies. Although the observed associations may reflect confounding by indication and greater underlying cardiovascular risk rather than a direct pharmacologic effect, it is essential to account for concomitant cardiovascular medications when interpreting the cardiac manifestations of severe hypothyroidism and underscore the need for careful contextualization.

This study has several limitations. First, its retrospective design precluded systematic echocardiographic reassessment at discharge, limiting within-patient comparisons before and after thyroid hormone replacement. Second, inclusion was restricted to patients who underwent TTE, as echocardiographic assessment was central to the study objectives. More than 60% of included cases occurred after routine bedside TTE became available in our ICUs (2020-2024), whereas nearly half of otherwise eligible patients were excluded because TTE was not routinely performed in earlier years. This reflects operational rather than clinical selection; although the magnitude of any resulting selection bias cannot be quantified, its impact on interpretation is likely limited. Third, although the etiology of hypothyroidism was undocumented in a subset of patients, autoimmune disease predominates in our geographic region, and etiologies with potential cardiotoxic effects were excluded. Finally, as an observational study, causal inferences cannot be established.

In conclusion, severe hypothyroidism and myxedema coma were frequently associated with LV dysfunction, including impaired myocardial deformation, reduced ejection fraction, and abnormal diastolic function. Cardiovascular medication exposure—particularly β-blockers and ACEis/angiotensin receptor blockers—was associated with worse echocardiographic indices; however, given the observational design, these relationships most likely reflect confounding by indication and underlying clinical context rather than causal effects. Despite the high prevalence of myocardial abnormalities, short-term mortality was low. Prospective studies incorporating standardized, longitudinal imaging before and after thyroid hormone replacement are needed to define reversibility, long-term outcomes, and cardiovascular risk stratification in this population.

## Data Availability

Restrictions apply to the availability of some or all data generated or analyzed during this study to preserve patient confidentiality or because they were used under license. The corresponding author will, on request, detail the restrictions and any conditions under which access to some data may be provided.

## Abbreviations

ACEi: angiotensin-converting enzyme inhibitor
CMRI: cardiac magnetic resonance imaging
EC: excitation-contraction
GLS: global longitudinal strain
ICU: intensive care unit
IQR: interquartile range
LV: left ventricular
LVDF: left ventricular diastolic function
LVEF: left ventricular ejection fraction
RV: right ventricular
SD: standard deviation
STE: speckle-tracking echocardiogram
TH: thyroid hormone
TSH: thyroid-stimulating hormone
TTE: transthoracic echocardiography
